# Determination of prenatal exposure to parabens and triclosan and estimation of maternal and fetal burden

**DOI:** 10.1016/j.toxrep.2021.03.030

**Published:** 2021-04-02

**Authors:** Vasiliki Karzi, Manolis N. Tzatzarakis, Eleftheria Hatzidaki, Ioanna Katsikantami, Athanasios Alegakis, Elena Vakonaki, Alexandra Kalogeraki, Elisavet Kouvidi, Pelagia Xezonaki, Stavros Sifakis, Apostolos K. Rizos

**Affiliations:** a Laboratory of Toxicology, Medicine School, University of Crete, Heraklion, Crete, GR-70013, Greece; b Department of Neonatology & NICU, University Hospital of Heraklion, Crete, GR-71500, Greece; c Department of Pathology-Cytopathology, Medical School, University of Crete, Heraklion, Crete, GR-70013, Greece; d Genesis Genoma Lab, Genetic Diagnosis, Clinical Genetics & Research, Athens, GR-15232, Greece; e Mitera Maternity Hospital, Heraklion, Crete, GR-71201, Greece; f Department of Chemistry, University of Crete and Foundation for Research and Technology - Hellas (FORTH-IESL), Heraklion, Crete, GR-71003, Greece

**Keywords:** Parabens, Triclosan, Urine, Amniotic fluid, Pregnant women, LC–MS

## Abstract

**Background:**

Parabens (PBs) and triclosan (TCS) are generally used as antimicrobials mostly in personal care products. Their wide prevalence in daily products raised an acute need for the biomonitoring of these contaminants and the investigation of possible health impacts.

**Material and methods:**

In this study we aimed to quantitatively determine PBs and TCS levels in urine and amniotic fluid samples using a liquid chromatography – mass spectrometry system (LC–MS). Ninety nine (99) pregnant women took part in this research. The samples were collected during the amniocentesis in the early second trimester of their pregnancy. Women of all ages, education, household income and profession were selected. The exposure and the burden of pregnant women and their infants were also evaluated.

**Results:**

The most prevalent compound in urine, among the analyzed, was TCS with 74.7 % positive samples while in amniotic fluid methyl paraben (MePB) with 21.2 % positive samples. MePB was detected at higher concentrations in urine (mean: 378.5 ng/mL) followed by TCS (mean: 55.3 ng/mL), ethyl paraben (EtPB) (mean: 23.2 ng/mL) and butyl paraben (BuPB) (mean: 2.3 ng/mL) while benzyl paraben (BePB) was not detected in any urine sample. Concentrations in amniotic fluid samples were much lower. In particular, the mean concentrations were 6.6 ng/mL for MePB, 9.2 ng/mL for EtPB, 0.4 ng/mL for BuPB, 0.6 ng/mL for BePB and 1.8 ng/mL for TCS. The detected levels of all analytes in urine were correlated with those in amniotic fluid but no statistically significant results arose (p >n0.05). Negative associations were observed between amniotic fluid levels of MePB and maternal age (p = 0.05) while both urinary and amniotic levels of TCS were correlated with maternal BMI (p = 0.04). Somatometric characteristics of the infants showed no statistical significant associations with the detected levels of PBs and TCS.

**Conclusion:**

This study indicated a strong/possible association between exposure of pregnant women to TCS and higher/lower maternal body weight gain during pregnancy. The same trend was observed between amniotic fluid MePB levels and maternal age. However, no statistically significant associations were observed between neonatal somatometric characteristics or health status and PBs and TCS levels.

## Highlights

• PBs and TCS levels were higher in maternal urine than amniotic fluid.
• TCS urine levels were associated with maternal weight gain during pregnancy.
• MePB amniotic fluid levels were associated with maternal age.
• No associations were observed between detected levels and infants’ somatometric characteristics and health condition.

## Graphical abstract

**Figure:**
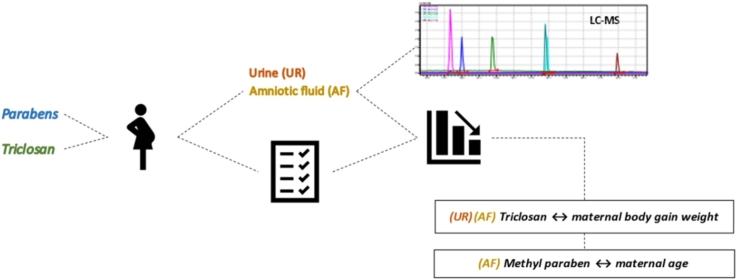

## 1 Introduction

Parabens (PBs) and triclosan (TCS) are phenolic compounds considered as antimicrobial agents with a wide range of activity. The main route of human exposure is personal care products (PCPs), such as deodorants, shower gels, shampoos, body creams and lotions [1]. PBs are also used in pharmaceuticals [2] and paper products, like baby napkins and paper food packages [3], while TCS in paper products and oral hygiene products like toothpastes and mouthwashes [4]. Studies have shown that PBs and TCS are present in wastewater discharges, entering this way the aquatic environment [1,4]. This fact raises concerns about the actual burden of plant and animal organisms and therefore human’s burden.

European Union has notified specific guidelines regarding the presence of PBs and TCS in consumer products. In particular, the maximum permissible concentration of TCS in cosmetics is 0.3 % [5], while the corresponding percentage for one paraben (PB) is 0.4 % and for all PBs 0.8 % [6]. USA and Canada have suggested the same limitations [7] while in Japan the upper limit for PBs in cosmetics has been set to 1.0 % [8].

Despite the limitations, the widespread use of PBs and TCS has resulted in the long-termhuman exposure which has raised concerns about the possible health impacts in the human body. PBs and TCS have been characterized as endocrine disruptors (EDs) as several studies have indicated that they affect the proper action of hormones and therefore the proper function of the endocrine system, leading to several health problems. Until now, studies indicate that exposure to PBs is correlated with male (even to the descendants) and female reproductive problems, breast cancer, increased chance of developing obesity, potential genotoxicity, increased sensitivity to allergens and neurotoxic effects [9]. Similar health effects have also been reported for TCS, except for neurotoxicity. Particularly, male and female reproductive problems [10,11] as well as cause and enhance of sensitivity to allergens [12,13] have been correlated with TCS exposure.

The burden of general population usually is estimated by hair analysis [[14], [15], [16]]. The last two decades, biomonitoring studies focus on estimating fetus burden by analyzing amniotic fluid [[17], [18], [19]] and meconium [20]. However, there are very few studies on monitoring of PBs and TCS in amniotic fluid [21,22] and only one of them correlates prenatal exposure with maternal urine and amniotic fluid levels [23]. PBs are metabolized in liver and intestine and are excreted in urine as metabolites of glycine, glycuronide and sulfonide [24], while they are transferred to the amniotic fluid through placental passive diffusion [21]. TCS is absorbed by the gastrointestinal tract and excreted in urine in its glycuronated and sulfonated form [25]. It is also transferred to the amniotic sac through the placenta [26]. The aim of our study was to determine methyl paraben (MePB), ethyl paraben (EtPB), butyl paraben (BuPB), benzyl paraben (BePB) and TCS levels in urine and amniotic fluid samples of pregnant women using a liquid chromatography – mass spectrometry system. Biomonitoring data were correlated with maternal somatometric and socio-economic characteristics, health problems, nutritional and lifestyle habits as well as infants’ developmental parameters and health condition. The impacts of prenatal exposure on infant development and pregnant women’s health were evaluated.

## 2 Material and methods

### 2.1 Materials

MePB, EtPB, BuPB, BePB, ethylacetate (High Pressure Liquid Chromatography (HPLC) grade), hydrochloric acid (≥37 %), ammonium acetate (BioXtra, ≥98 %) and solid phase extraction (SPE) cartridges C18 (100 mg) were purchased by Sigma – Aldrich (St. Louis, MO, USA). TCS (100 %) was obtained by Honeywell–Fluka (Seelze, Germany), methanol (LC-MSgrade) and acetonitrile (Liquid Chromatography - Mass Spectrometry (LC–MS) grade) by Honeywell–RiedeldeHaën (Seelze, Germany) and phenobarbital-d5 used for internal standard by Isotec Inc. (Miamisburg, OH, USA). Phosphate buffer saline was purchased from FlukaBiochemika (Steinheim, Switzerland) and β-glucuronidase enzyme from *Escherichia coli* from Roche Diagnostics (Mannheim, Germany). Ultrapure water was produced by Merck’s Direct-Q 3UVwater purification system (Darmstadt, Germany).

### 2.2 Study group

Sampling took place at the “Mitera” Maternity Hospital in Heraklion, Crete. Maternal urine and amniotic fluid samples were collected from ninety nine (99) pregnant women during the amniocentesis in early second trimester of their pregnancy. The amniocentesis procedure was scheduled by the doctor due to one (or more) of the following reasons; the maternal age, the maternal medical/obstetric history and the health condition of both mother and fetus. In specific, in Greece, it is almost obligatory to conduct amniocentesis on pregnant women over the age of 35. Also, in case that the mother, or fetus, had presented any health problem (e.g. genetic abnormality) either in previous or the current pregnancy, amniocentesis was necessary to ensure the health of both mother and fetus.Before sampling procedure, all participating women were informed about the research and asked to sign participation consent. Samples were collected in screw glass tubes and stored at −20 °C until analysis. This study was approved by the ethics committee of the University of Crete (43/22.11.2018).

### 2.3 Questionnaires’ data

During the sampling, the participants were asked to complete questionnaires about maternal demographic (e.g., age, education) and somatometric characteristics (e.g., height, weight, body mass index (BMI)) before and during pregnancy, nutritional (e.g., consumption of dairy products) and lifestyle habits (e.g., smoking, alcohol consumption), medical and obstetrical history and the frequency of personal care products. Six to twelve months after childbirth, additional information was collected regarding infants’ development (weight, length, head circumference (HC)) and health condition as well as birth type (normal or cesarean).

### 2.4 Amniotic fluid extraction

1 mL of each amniotic fluid sample was placed in glass screw tubes, since the samples had been centrifuged. Subsequently, 10 μl of β-glucuronidase enzyme was added to each sample to hydrolyze the conjugated (glucuronidized) compounds and 250 μl of 0.1 M phosphate buffer pH 6.5 to create the appropriate environment for the enzyme to act. The samples were incubated in a water bath at 37 °C for 12 h. After incubation, 100 μl of 2 M hydrochloric acid were added, followed by extraction with 2 mL of ethyl acetate. The samples were shaken for 20 min and then the extract, that is the organic phase, was transferred to glass evaporation tubes. This process was repeated twice, followed by evaporation to dryness under nitrogen flow, reconstitution of the evaporation residue in 100 μl of methanol and transfer to 2 mL vials (inside inserts) for LC–MS analysis.

### 2.5 Urine extraction

Similar procedure was followed for urine samples. 1 mL of each sample was placed (after centrifugation) in glass screw tubes and 10 μl of β-glucuronidase enzyme and 250 μl of 0.1 M phosphate buffer pH 6.5 were added. After overnight incubation at 37 °C, 100 μl of 2 M hydrochloric acid were added, followed by extraction with 2 mL of ethyl acetate three times. At this stage of the experimental procedure, an extra clean step was added using SPE cartridges. In particular, the extracts were evaporated to dryness and then reconstituted in 1 mL of buffer solution pH = 2. The cartridges were cleaned and activated using 2 mL of acetonitrile, 2 mL of acetorintrile:water 1:1 and 2 mL of water. Then, the samples were loaded and washed with 2 mL of water. After cartridges had been dried up, the analytes were extracted using 2 mL of acetorinitrile : ethyl acetate 1:1. Finally, the extracts were collected, evaporated to dryness under nitrogen flow, reconstituted in 100 μl of methanol and analyzed with LC–MS.

### 2.6 Instrumentation

A Shimadzu (Kyoto, Japan) liquid chromatography – mass spectrometry system (LC–MS 2010 EV model) equipped with an autosampler was used. Separation of the analytes was achieved using a Supelco Discovery column C18 (25 cm) (Sigma – Aldrich, St. Louis, MO, USA) at 30 °C stable oven temperature. A gradient of 5 mM ammonium acetate (solvent A) and acetonitrile (solvent B) were chosen for the analysis of PBs and TCS. The flow of chromatography was 0.6 mL/min and the total duration of the run for each sample was 25 min. Atmospheric pressure chemical ionization (APCI) combined with a quadrupole mass filter in a selected ion monitoring (SIM) negative mode were used for monitoring the aforementioned substances. In Table 1, the retention time (Rt) and the used *m/z* of each analyte are given.

**Table 1:** Parameters of the analysis.

	Rt (min)	Target ion (m/z)	Secondary ions (m/z)
MePB	13.07	151.05	194.0
EtPB	14.87	165.05	208.1
BuPB	17.87	227.1	287.15
BePB	17.93	193.05	236.05
TCS	22.0	286.95	289.0, 291.0
Internal Standard	12.47	236.05	–

### 2.7 Statistical methods

Measures of central tendency (mean, median, min, max) and measures of dispersion (standard deviation, interquartile range) were used according to the data normality for the continuous variables. Counts, percentages and proportions were applied for discrete data. The correlation of discrete variables was tested using Pearson's χ2 test, changes to paired measurements of discrete data were tested using McNemar for 2 × 2 tables or Mc Nemar-Bowker test for nxn tables. Pearson’s rho coefficient was used to estimate correlation of continuous and ordinal variables. Boxplots were used for representing ordinal and discrete data and scatterplots were used for examining correlation between continuous variables. IBM SPSS Statistics 24.0 was used for data analysis and a level of acceptance was set at p = 0.05 level.

## 3 Results

### 3.1 Methods validation

Analytical parameters of the applied methods were checked and evaluated for the target compounds. Standard solutions were prepared at concentrations 0, 25, 50, 100, 250 and 500 ng/mL and the obtained calibration curves showed that linearity (R^2^) was >0.99 for all compounds in both matrices. Linearity obtained from spiked samples at concentrations 0, 5, 10, 25, 50 and 100 ng/mL was similar with that of standard solutions (R^2^>0.99).

Recovery (%), accuracy (%) and inter day precision (%) were also calculated for both urine (three replicates, n = 3) and amniotic fluid (four replicates, n = 4) and found to be within the accepted values.

Limits of detection (LOD) were calculated using the signal to noise ratio (S/N); S/N > 3 for LOD. The calculated values were 0.07 ng/mL for MePB, 0.31 ng/mL for EtPB, 0.12 ng/mL for BuPB, 0.17 ng/mL for BePB and 0.73 ng/mL for TCS.

### 3.2 Questionnaires’ data

A total of 99 women participated in the study and the mean age was 35.2 ± 5.8 years ranging from 18.0 to 44.0 years. Reported BMI before pregnancy was 24.4 ± 5.6 Kg/m2 (range 16.0–45.4) and the corresponding at the time of amniocentesis was 25.9 ± 5.3 Kg/m2 (range 17.2–46.3) showing a statistically significant increase (p < 0.001) (mean DBMI: −1.5 ± 1.4).

Medical history of the participating women showed that thyroid problems were the most common (32.3 %), followed of allergic disorders/diseases (29.3 %), gynecological (24.2 %) and respiratory problems (7.1 %). Eighty-five mothers accepted to give additional information about infants’ development (6months – one year after birth) and possible health issues. Female neonates were 44 (51.7 %) and only 7 female neonates were born pre-term (<37 gestation weeks). Frequency of pre-term gestation did not differ between male and female neonates (p = 0.663). Female neonates presented a mean weight at birth 3048 ± 559 g which do not differ significantly from male neonates 2981 ± 670 g (p = 0.621). Similar findings were found for neonates’ length vs neonates’ gender (p = 0.790) with a mean length of 50.2 ± 2.9 cm and for neonates’ head circumference vs gender (p = 0.949) with a mean HC of 34.7 ± 1.5 cm. A total of 12 newborns 14.1 % suffered from a disease/disorder. The most common reported health issues were allergic disorders (10.6 %) followed by respiratory problems (2.4 %) and genital abnormalities (1.2 %) (Table 2).

**Table 2:** Somatometriccharacteristics of newborns (weight, length and head circumference) and reported health problems.

		N	%N	Mean	SD	p
Weight (gr)	Female	44	51.8	2981	670	0.621
	Male	41	48.2	3048	559	
	Total	85		3013	616	
Length (cm)	Female	39	50	50.3	2.6	0.790
	Male	39	50	50.2	3.3	
	Total	78		50.2	2.9	
Head circumference (cm)	Female	39	52.7	34.7	1.7	0.949
	Male	35	47.3	34.7	1.1	
	Total	74		34.7	1.5	
Health problems	Allergies	9	10.6			
	Respiratory problems	2	2.4			
	Genital abnormalities	1	1.2			
	Total	12	14.1			

### 3.3 PBs and TCS levels in urine and amniotic fluid

Descriptive statistics of measured PBs (MePB, EtPB, BePB, BuPB) and triclosan (TCS) are given in Table 3. In amniotic fluid samples the higher detection rate was observed for MePB (21.2 %) with mean concentration 6.6 ± 5.7 ng/mL. Among all the rest compounds, only TCS showed a detection rate slightly over 5.0 % and only one sample was positive for each of BePB (0.6 ng/mL) and EtPB (1.3 ng/mL). In urine samples, BePB was not detected in any sample while for the other PBs the detection rate ranged from 8.1 % (EtPB) to 64.6 (MePB). TCS was detected in 74.7 % of the samples.The minimum concentration was observed for TCS (0.7 ng/mL) and the maximum for MePB (3501.3 ng/mL).

**Table 3:** Descriptive statistics of levels of PBs and TCS in urine and amniotic fluid.

		Positive (%)	Mean (ng/mL)	SD	Median (ng/mL)	Range (ng/mL)
Amniotic fluid (ng/mL)	MePB	21.2	6.6	5.7	5.0	0.1 – 18.8
	EtPB	1.0	1.3	–	1.3	1.3
	BePB	1.0	0.6	–	0.6	0.6
	BuPB	2.0	0.4	0.3	0.4	0.2 – 0.6
	TCS	5.1	1.8	0.7	2.0	0.9 – 2.4
Urine (ng/mL)	MePB	64.6	378.5	664.3	59.6	5.3 – 3501.3
	EtPB	8.1	23.2	30.8	8.5	0.8 – 81.7
	BePB	0.0	–	–	–	–
	BuPB	13.1	2.3	1.7	1.9	1.2 – 7.6
	TCS	74.7	55.3	125.9	10.0	0.7 – 615.5

Urinary levels ranged from 5.3 to 3501.3 ng/mL (mean: 378.5 ng/mL) for MePB, from 0.8 to 81.7 ng/mL (mean: 23.2 ng/mL) for EtPB, from 1.2 to 7.6 (mean: 2.3 ng/mL) for BuPB and from 0.7 to 615.5. ng/mL (mean: 55.3 ng/mL) for TCS. The big range of MePB and TCS is attributed to the fact that these two substances are the most common used antimicrobials in personal care products. Similar ranges have been observed in previous studies and other biological matrices, too [27]. In amniotic fluid samples the corresponding levels were 0.2–18.8 ng/ml (mean: 6.6 ng/mL) for MePB, 0.2 – 0.6 ng/mL (mean: 0.4 ng/mL) for BuPB and 0.9–2.4 ng/ml (mean: 1.8 ng/mL) for TCS.

**Fig. 1:**
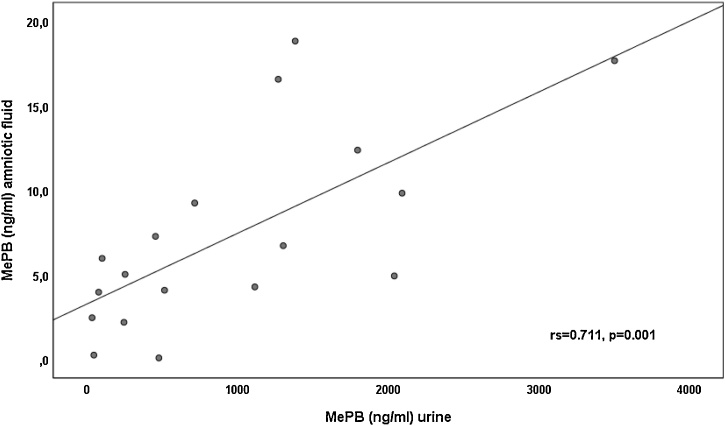
Correlation of MePB levels in urine and amniotic fluid (rs = 0.71, p = 0.01).

Due to the small prevalence of positive samples for PBs and TCS, correlation between them was examined only for MePB and the estimated rs was 0.711 (p < 0.001) (Fig. 1).

### 3.4 Correlation of monitoring results with questionnaires data

**Fig. 2:**
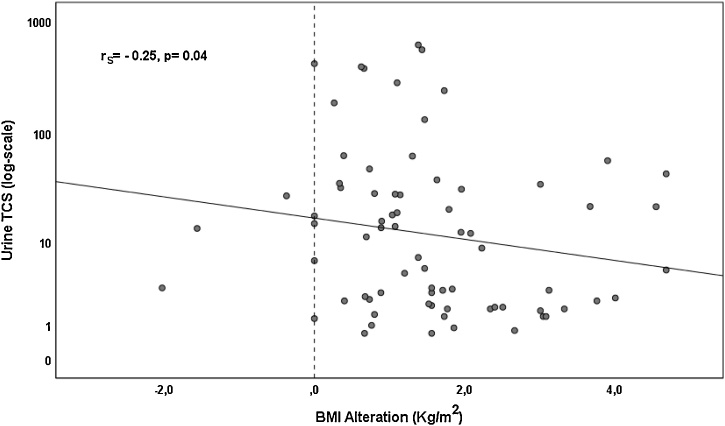
Correlation of urine TCS levels (log scale) and mothers’ BMI alteration (kg/m^2^).

**Fig. 3:**
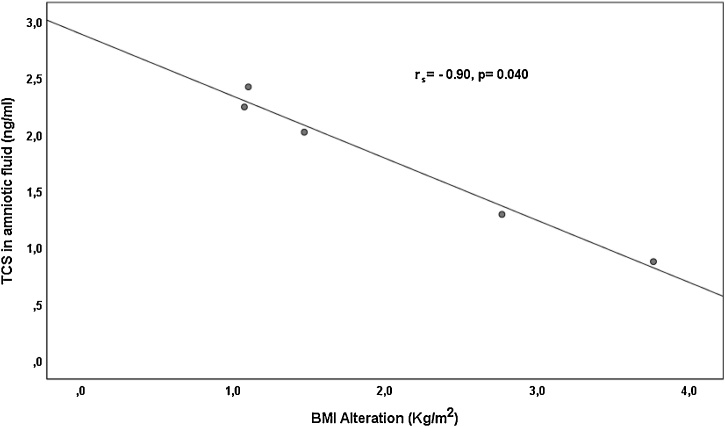
Correlation of amniotic fluid TCS levels (ng/mL) and mothers’ BMI alteration (kg/m^2^).

Detected levels of PBs and TCS in urine and amniotic fluid were correlated with the somatometric characteristics of women. Correlation results are shown in Table 4. It can be seen that there were negative associations (p < 0.100) between amniotic levels of MePB and mother’s age (rs=−0.43, p = 0.05). Urinary and amniotic levels of TCS were correlated (p < 0.100) with mother’s BMI alteration (rs=−0.25, p = 0.04 and rs=−0.90, p = 0.040, respectively) (Figs. 2 & 3 ).

**Table 4:** Correlationof PBs and TCS levels in urine and amniotic fluid with maternal somatometrics.

			Age	Weight before pregnancy	Weight during pregnancy	Dweight	BMI before pregnancy	BMI during pregnancy	DBMI
Amniotic fluid	MePB	Rs	−0,428	−0,355	−0,313	0,046	−0,364	−0,405	0,086
		p	0,053	0,114	0,167	0,842	0,105	0,068	0,712
	TCS	Rs	0,103	−0,564	−0,700	−0,975	−0,700	−0,700	−0,900
		p	0,870	0,322	0,188	0,005	0,188	0,188	0,037
Urine	MePB	Rs	−0,094	−0,069	−0,092	−0,044	−0,109	−0,129	−0,041
		p	0,462	0,588	0,472	0,733	0,392	0,315	0,751
	EtPB	Rs	0,252	−0,167	−0,156	−0,521	−0,381	−0,524	−0,503
		p	0,548	0,693	0,713	0,185	0,352	0,183	0,204
	BuPB	Rs	−0,003	0,242	0,104	−0,209	0,197	0,223	−0,212
		p	0,993	0,426	0,747	0,515	0,519	0,487	0,507
	TCS	Rs	−0,029	0,116	0,067	−0,215	0,041	−0,003	−0,245
		p	0,808	0,324	0,574	0,068	0,728	0,977	0,037

## 4 Discussion

In the current study we aimed to determine PBs and TCS levels in urine and amniotic fluid samples from pregnant women. Maternal and fetal exposure as evaluated from biomonitoring data in urine and amniotic fluid, respectively, were associated with maternal demographic and somatometric characteristics as well as daily habits and medical history. Infants’ somatometric characteristics and health condition were also correlated with the found levels.

The target compounds were mostly present in urine samples with % positivity ranging from 8.1 to 74.7 % for urine samples and from 1.0 to 21.2 % for amniotic fluid samples. MePB had the higher detection frequency (21.2 %) and mean concentration level (6.6 ng/mL) among the detected compounds in amniotic fluid. MePB presented also high detection frequency (64.6 %) in urine samples but TCS frequency was even higher (74.7 %). The higher concentration levels in urine were observed for MePB with mean value 378.5 ng/mL followed by TCS with mean concentration 55.3 ng/mL. BePB was not detected in urine while in amniotic fluid there was only one positive sample (0.6 ng/mL). Additionally, EtPB and BuPB presented very low detection rates in amniotic fluid (1.0 % and 2.0 %, respectively). This high difference of detection rates and concentration levels supports existing views that PBs and TCS has lower biotransformation/metabolism in amniotic fluid than in urine which leads to insignificant accumulation potential [21,26,28].

Until now there are only three published studies on biomonitoring of PBs and TCS in amniotic fluid. The detected concentrations in the present study are similar with the levels reported in the literature [[21], [22], [23]]. On the other hand, the biomonitoring studies for PBs and TCS in urine are plenty and our results are similar with the levels in literature. Jamal and co-authors reported median concentration 14.08 ng/mL (1.64–65.78 ng/ml) for TCS, 242.51 ng/mL (9.67–744.56 ng/ml) for MePB, 2.0 ng/mL (<0.25–101.19 ng/mL) for EtPB and 1.42 ng/mL (<0.25–7.43 ng/mL) in urine samples from 189 Iranian pregnant women [29]. Similar values were published by Hajizadeh and co-authors who studied the PBs burden in urine samples from 95 Iranian pregnant women [30]. In particular, the median concentration was 87.0 ng/mL (1.01–955 ng/ml) for MePB, 9.64 ng/mL (<0.015–175 ng/mL) for EtPB and 8.57 ng/mL (<0.016–97.9 ng/mL) for BuPB (Table 5).

**Table 5:** PBs and TCS urinary levels (ng/mL) during pregnancy reported in the literature.

	N	Country	Mean	Median	Range	Reference
**TCS**	99	Greece	50.6	5.4	0.3 – 615.5	*Current study*
	189	Iran	14.69	14.08	1.64 – 65.78	*Jamal et al., 2020* [29]
	10	Israel	57.4		<LOD – 342.9	*Zhong et al., 2019* [61]
	377	USA		16	<2.4 – 1501	*Etzel et al., 2017* [41]
MePB	99	Greece	378.5	59.6	5.3 – 3501.3	*Current study*
	189	Iran	253.05	242.51	9.67 – 744.56	*Jamal et al., 2020* [29]
	95	Iran	142	87.0	1.01 – 955	*Hajizadeh et al., 2020* [30]
	13	Canada	94.86		0.42 – 1751.18	*Fisher et al., 2017* [62]
	12	USA		104	9.97 – 1182	*Messerlian et al., 2017* [63]
EtPB	99	Greece	23.2	8.5	0.8 – 81.7	*Current study*
	189	Iran	2.69	2.0	<0.25 – 101.19	*Jamal et al., 2020* [29]
	95	Iran	24.9	9.64	<0.015 – 175	*Hajizadeh et al., 2020* [30]
	13	Canada	15.13		0.01 – 398.03	*Fisher et al., 2017* [62]
	12	USA		9.70	0.77 – 84.1	*Messerlian et al., 2017* [63]
BuPB	99	Greece	2.3	1.9	1.2 – 7.6	*Current study*
	189	Iran	2.01	1.42	<0.25 – 159.86	*Jamal et al., 2020* [29]
	95	Iran	14.8	8.57	<0.016 – 97.9	*Hajizadeh et al., 2020* [30]
	13	Canada	3.28		0 – 166.92	*Fisher et al., 2017* [62]
	12	USA		1.09	0.04 – 10.3	*Messerlian et al., 2017* [63]

Philippat and co-authors correlated the levels of PBs and TCS in maternal urine with those in amniotic fluids, and found that urine and amniotic fluid followed the same trend. For instance, women with high urinary MePB and TCS concentrations presented also high levels in amniotic fluid [23]. Although the positivity of the target compounds in our study was <25 % for amniotic fluids, compounds with high detection rate in urine samples (MePB 64.6 % and TCS 74.7 %) provided the higher detection rates in amniotic fluids (MePB 21.2 % and TCS 5.1 %).

According to the literature, urinary PBs levels generally show the following trend MePB > EtPB > BuPB > BePB. The same distribution/order is observed in our study, too, and the respective mean concentrations are 378.5 ng/mL for MePB, 23.2 ng/mL for EtPB, and 2.3 ng/mL for BuPB, while BePB was not detected in any sample. Shekhar and co-authors reported a similar trend for amniotic fluids, specifically MePB > TCS > EtPB > BuPB, which is also observed in our results [22]. This fact has been confirmed by Song and his team who found that placental transfer rates are increased when the molecular weight of PBs is small [21]. This means that PBs of small molecular weightsuch as MePB, tend to be transferred more easily through the placenta to the amniotic sac. The aforementioned detection order was not observed in a recent study conducted in the Republic of Korea, in which higher EtPB concentrations were detected in urine samples. This fact was attributed to the authorization granted by the Korean Ministry of Food and Drug Administration to use EtPB as a preservative in food stuff [31].

Correlation of maternal demographic characteristics with the detected levels showed no statistically significant results except for maternal age which was negatively correlated with MePB levels in amniotic fluid. There is no such association reported in literature however Ashrap and co-authors found that urinary PBs levels were higher in elderly pregnant women [32]. The same association was observed for TCS, too [33,34]. Studies have indicated that PBs and TCS levels are higher in women with lower education level [35], but in the current study there was no such conclusion. PBs levels have found to be higher when the household income of pregnant women is high, but in the case of TCS there is a controversy concerning the potential of being positively [36,37] or negatively [38] correlated with the household income with the latter opinion being predominant.

In the current study, urinary TCS levels were higher when small BMI alteration during pregnancy was observed. Li and co-authors also indicated that BMI is possibly associated with TCS [33]. Additionally, high exposure to PBs has been associated with increased body weight expressed as BMI [30,39]. It is worth mentioning that higher urinary levels of PBs were detected in pregnant women with increased gain weight rate and this relation was statistically significant [40], raising questions concerning the role that fat plays in PBs accumulation.

Infants’ somatometric characteristics have been previously correlated with both TCS and PBs. Previous studies have indicated that birth length, weight and head circumference tend to be smaller when TCS levels are high [29,41,42] while in the case of PBs the conclusions are not so clear. Wu and co-authors reported that when urinary PBs levels were high, birth weight in boys was also high but girls’ birth weight was low [43]. Jamal et al. found heavier birth weight in cases that BuPB was high and also larger head circumference in cases that MePB and BuPB were high [29]. However, there are studies that cited inverse associations between birth weight and MePB [44] and EtPB [43] as well as between MePB and head circumference [45]. As regards birth length, Etzel et al. reported that is inversely associated with PBs [41].

One of the most debatable issues of PBs and TCS exposure is the risk that may arise for both the mother and the infant. There are animal studies that claim preterm birth or miscarriage after animals’ exposure to PBs and/or TCS [46,47]. Although, this refers to animals, which means to a little different organisms, this fact raises concerns about human pregnancies and the effects that exposure to these substances would cause. It has already been proved that both TCS and PBs are inversely associated with thyroid hormones (T3, T4, TSH) [[48], [49], [50]], a fact that is a little bit concerning considering that these hormones define also infant’s hormones system. Except for the aforementioned, PBs are also negatively associated with maternal blood pressure [51] and EtPB has been strongly associated with preterm birth [47]. TCS is associated with maternal blood pressure [52]. Li and co-authors observed that there is a high risk of gestational diabetes mellitus when overweight/obese pregnant women are exposed to PBs [33] while Kang and co-authors found significant associations between MePB and EtPB and oxidative biomarkers [53].

There are several studies that correlate children exposure to PBs and TCS with allergies and asthma incidents. However, when it comes to maternal exposure and infants’ burden only PBs are correlated with increased asthma rates [54]. The increased maternal progesterone and estradiol levels associated with TCS and PBs [55] exposure raise concerns about the outcome that they may have in infant’s reproductive system. There is strong evidence that prenatal exposure to PBs and/or TCS leads to neurobehavioral problems in descendants. In specific, lower intellectual functioning, poorer verbal and memory skillsandchanges in locomotor activity have been observed in children that were prenatally exposed to these substances [56,57]. Animal experiments have also shown that gestational exposure to TCS is related toanxiety-like behaviors, muscle strength and adverse effects in brain tissue [58], while exposure to PBs negatively affects mitochondrial function in testicles which leads to ROS (reactive oxygen species) production and modulation of antioxidant system in different organs [59].It is worth pointing out that Michels and co-authors found that exposure TCS leads to reduction of infants’ telomeres length, an indicator of biological aging [60].

## 5 Conclusions

To the best of our knowledge, the current study is one of the very few, that aimed to correlate maternal and fetal burden to PBs and TCS. Also, this is of the very few studies that tried to assess the exposure of Greek women to these substances. The importance of this study lies in the fact that PBs and TCS are endocrine disruptors strongly correlated with fetus developmental problems and subsequent postnatal problems in growth. The study population consisted of women residents of Crete; a big Greek island which lands away from the mainland. The participants originated from all social layers with different incomes, education and professions. This way, we tried to simulate the exposure of Greek women using a sample size of 99 women. Although urine and amniotic fluid levels of PBs and TCS were not associated, significant associations arose between the detected levels and both maternal age and BMI. The correlation between urine – amniotic fluid levels and neonatal somatometric characteristics as well as neonatal health status did not result in statistically significant conclusions. This fact may be attributed to the low prevalence of the target compounds in the analyzed samples. Nevertheless, the detection of these compounds in maternal urine and amniotic fluid raises concerns about the real risk to both mothers and fetuses; hence further studies must be conducted in order to elucidate it. One fact that must be taken into consideration is the combined exposure to these substances and their potential synergistic toxicity [64] as this is a new era in toxicology that must be explored.

## Author statement

Vasiliki Karzi: Investigation, Formal analysis, Writing-review & editing; Manolis M. Tzatzarakis: Conceptualization, Supervision, Methodology; Eleftheria Hatzidaki: Resources, Validation; Ioanna Katsikantami: Investigation; Athanasios Alegakis: Formal analysis, Data curation; Elena Vakonaki: Methodology; Alexandra Kalogeraki: Resources; Elisavet Kouvidi: Resources; Pelagia Xezonaki: Resources; Stavros Sifakis: Resources, Validation; Apostolos K. Rizos: Conceptualization, Supervision.

## Declaration of Competing Interest

The authors report no declarations of interest.
